# Advances in the Identification Methods of Food-Medicine Homologous Herbal Materials

**DOI:** 10.3390/foods14040608

**Published:** 2025-02-12

**Authors:** Yuying Jiang, Shilei Wei, Hongyi Ge, Yuan Zhang, Heng Wang, Xixi Wen, Chunyan Guo, Shun Wang, Zhikun Chen, Peng Li

**Affiliations:** 1 Institute for Complexity Science, Henan University of Technology, Zhengzhou 450001, China; jiangyuying11@163.com; 2 School of Artificial Intelligence and Big Data, Henan University of Technology, Zhengzhou 450001, China; 3 Key Laboratory of Grain Information Processing and Control, Ministry of Education, Henan University of Technology, Zhengzhou 450001, China; wsl_20011213@163.com (S.W.); gehongyi2004@163.com (H.G.); zy_haut@163.com (Y.Z.); wh843754374@163.com (H.W.); wenxixi991214@163.com (X.W.); gcy20001104@163.com (C.G.); ws_0702@163.com (S.W.); czk15237093431@163.com (Z.C.); 4 College of Information Science and Engineering, Henan University of Technology, Zhengzhou 450001, China

**Keywords:** Food-Medicine homologous, herbal materials, chromatography–mass spectrometry coupling, hyperspectral imaging, near-infrared spectroscopy, terahertz spectroscopy, DNA barcoding

## Abstract

As a key component of both traditional medicine and modern healthcare, Food–Medicine Homologous Herbal Materials have attracted considerable attention in recent years. However, issues related to the quality and authenticity of medicinal materials on the market often arise, not only compromising their efficacy but also presenting potential risks to consumer health. Therefore, the establishment of accurate and efficient identification methods is crucial for ensuring the safety and quality of Food-Medicine Homologous Herbal Materials. This paper provides a systematic review of the research progress on the identification methods for Food-Medicine Homologous Herbal Materials, starting with traditional methods such as morphological and microscopic identification, and focusing on the applications of modern techniques, including biomimetic recognition, chromatography, mass spectrometry, chromatography–mass spectrometry coupling, hyperspectral imaging, near-infrared spectroscopy, terahertz spectroscopy, and DNA barcoding. Moreover, it provides a comprehensive analysis of the fundamental principles, advantages, and limitations of these methods. Finally, the paper outlines the current challenges faced by identification methods and suggests future directions for improvement, aiming to offer a comprehensive technical perspective on identifying Food-Medicine Homologous Herbal Materials and foster further development in this field.

## 1. Introduction

Food-Medicine Homologous Herbal Materials refer to plants or plant parts that are edible and possess established traditional medicinal value [1]. The *Huangdi Neijing*, the earliest medical classic in China, includes numerous dietary therapy formulas [2]. Increasing research indicates that Food-Medicine Homologous Herbal Materials confer substantial health benefits, including antioxidant [3], anti-aging [4], modulation of gut microbiota balance [5,6,7], reduction in blood pressure [8], blood sugar [9], blood lipids [10], and uric acid levels [11], inhibition of tumor growth [12], anticancer effects [13], antibacterial properties [14], regulation of angiogenesis [15,16], cardiovascular protection [17], and alleviation of alcohol-induced liver injury [18], among others. Food-Medicine Homologous Materials in this context are those developed based on traditional Chinese dietary therapy concepts, utilizing medicinal and edible Chinese herbs as raw materials. These products can be categorized into general homology-based foods and herbal health products, depending on the properties of the medicinal materials and the target consumer groups. General homology-based foods are made with medicinal and edible substances, primarily to provide nutrition or enhance flavor rather than for therapeutic purposes. Examples include ginger [19], cinnamon [20], and Sichuan pepper [21], which can be directly used as seasonings or incorporated as extracts to enhance aroma and taste while offering potential medicinal value. Herbal health products, on the other hand, are formulated based on traditional Chinese medicine (TCM) theories and are designed to promote overall health [22]. In contrast, homology-based medicinal products focus on the treatment of diseases or symptom relief. These herbal components are typically subjected to rigorous screening, purification, and standardization, with clearly defined dosage guidelines to ensure both efficacy and safety.

**Figure 1:**
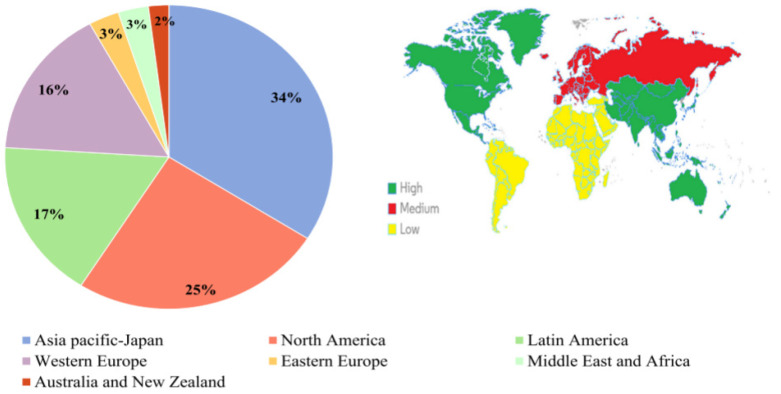
Geographical distribution of dietary supplements market and level of DS market growth worldwide (High, Medium, and Low) [26].

With the accelerating pace of modern life, people’s health awareness has gradually increased, and their attention to diet has steadily grown. A growing number of individuals are exploring how to regulate their bodies through diet and use “food therapy” as an alternative to “drug therapy”. Over the past two decades, the prevalence of health foods derived from Food-Medicine Homologous Materials has significantly increased. In fact, the sales revenue of this market has been continuously rising and, more importantly, the variety of available products has also expanded. Consumers now have access to a vast array of products, brands, and formulations through various marketing channels [23]. It is estimated that, in 2021, the global market for Food-Medicine homologous health foods was valued at approximately $152 billion. According to the latest report from Statista, this value is projected to reach $300 billion by 2028 [24]. The global market for these products is characterized by sustained sales growth, reinforcing the belief that they are an integral part of people’s diets worldwide [25]. The geographical distribution of the global Food-Medicine homologous health food market varies significantly, with six major regions identified (Figure 1) [26]. The United States, Europe, and Japan hold the largest market shares, followed by Asia, Australia, and Oceania, all of which indicate continuous market expansion [25]. In contrast, the Middle East and Africa have witnessed a surge in sales of these health products, with South Africa remaining the most significant market in the region [27].

**Figure 2:**
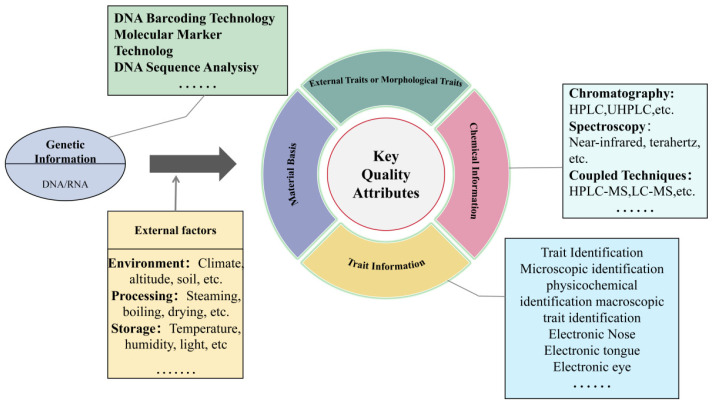
Key Quality Attributes of Herbal Medicine.

Since the late 1980s, China has initiated research and development regarding the ‘List of Items that are Both Food and Medicinal Materials’. The list was first introduced in 1987 and has undergone ten revisions, expanding from an initial 33 items to 106 items by 2024 [28], demonstrating China’s continued focus on and expansion of Food-Medicine homologous varieties. Additionally, other countries have also established relevant regulations or documents on this matter. Additionally, many other countries have established regulations or guidelines related to food products with medicinal and dietary homology. For example, the Russian Federation follows the 11th edition of the Soviet State Pharmacopoeia, which includes 83 monographs on medicinal plants, 51 of which are also listed in the European Pharmacopoeia and have been extensively studied [29]. Japan was the first to propose the concept of “functional foods” and revised its Nutrition Improvement Act in 1991, classifying functional foods under the category of special-purpose foods [30]. The U.S. Food and Drug Administration (FDA) mandates that dietary supplement labels must not include terms like “treatment” or “disease”, but they may feature approved claims, such as health claims, nutrient content claims, and structure/function claims [31]. In May 2020, the U.S. National Institutes of Health (NIH) announced its 2020–2030 Strategic Plan for NIH Nutrition Research, which identified reducing the burden of disease in clinical care through the “Food as Medicine” initiative as one of its four strategic priorities. In the European Union, Directive 2002/46/EC defines dietary supplements as “foodstuffs intended to supplement the normal diet, which are concentrated sources of nutrients or other substances with a nutritional or physiological effect, alone or in combination” [32]. Researchers, both internationally and domestically, have extensively validated the safety of “medicinal and edible” foods through ongoing exploration and verification [33]. Figure 2 illustrates the key quality attributes of medicinal and edible materials with homology, integrating five critical aspects: genetic information, external environment, material basis, chemical properties, and performance characteristics. It also briefly summarizes the methods employed at each stage and the influencing factors encountered during cultivation, growth, and storage.

Artificial intelligence (AI) has emerged as a transformative technology with immense potential across various fields, including the identification and classification of Food-Medicine homologous herbal materials. Traditional methods of identifying medicinal plants often rely on manual observation, which can be time-consuming, labor-intensive, and prone to errors. One of the key advantages of AI-based methods is their ability to process large volumes of data and images rapidly, enabling high-throughput screening of Food-Medicine homologous plants [34]. The two critical subfields, machine learning (ML) and deep learning (DL), have played a significant role in automating plant species identification. By leveraging ML and DL technologies, automatic species recognition can be optimally achieved without the challenges associated with manual identification methods. Given the growing global emphasis on quality control, AI-driven intelligent identification methods provide robust technical support for ensuring the authenticity, safety, and standardization of Food-Medicine homologous herbal materials. The literature search for this paper was conducted using platforms such as Google Scholar, Web of Science, Elsevier, and the China National Knowledge Infrastructure (CNKI). A comprehensive search and selection of literature were performed by using keywords relevant to the manuscript’s topic.

## 2. Traditional Identification Methods for Food-Medicine Homologous Herbal Materials

### 2.1. Morphological Identification Method

Morphological identification inherits the traditional experience and methods of “distinguishing characteristics to determine quality”. It is simple and easy to perform, including the observation of appearance (such as color, shape, size, and surface texture), tactile texture (whether the medicinal material feels rough, smooth, or has specific textural differences), cross-sectional characteristics (examining the cross-section of the material, including its tissue structure, texture, and color variations), olfactory examination, and water tests to determine the species and quality of the medicinal material [35]. For instance, medicinal materials such as peppermint, angelica, and patchouli [36] can be identified by their distinctive and characteristic aroma. The water test involves placing the medicinal material in water and observing the unique phenomena that occur, which reveal its essential characteristics. For example, saffron turns golden yellow after soaking in water, the water becomes yellow, and the flowers unfold in a straight line, gradually taking on a trumpet-like shape [37]. *Fritillaria cirrhosa* was first documented in the Shennong Bencao Jing, classified as a medium-grade medicinal herb. Renowned as the “sacred medicine for relieving coughs”, it has a long history of use as an expectorant and antitussive agent [38]. In recent years, excessive harvesting has led to a continuous decline in its wild resources. Li et al. [39] utilized SPSS 20.0 software to conduct a bivariate correlation analysis of the height, diameter, and color difference of *Fritillaria cirrhosa* with its total alkaloid content. The results showed a highly significant negative correlation between height and total alkaloid content, as well as a negative correlation between diameter and total alkaloid content. This indicates that the smaller the size of *F. cirrhosa*, the higher its total alkaloid content, which fully supports the traditional view that the smaller variety, known as Songbei, is considered the best. In traditional Vietnamese medicine, *Panax vietnamensis* is widely used to treat neurological and cardiovascular diseases and exhibits anti-tumor, anti-aging, and anti-stress properties [40]. Extensive research on its chemical composition and pharmacological activities has been conducted domestically and internationally, establishing it as one of the most valuable ginseng species worldwide [41]. The distribution of *P. vietnamensis* overlaps significantly with that of *Panax notoginseng*, and its plant morphology is highly similar to that of *P. notoginseng*, *P. ginseng*, and *P. quinquefolius*. As a result, its medicinal materials are often easily confused with those of related species. Cheng et al. [42] conducted detailed observations and descriptions of various batches of *Panax vietnamensis* and its easily confused medicinal plants, focusing on the morphology of the source plants and the dried medicinal parts, including shape, size, surface color, cross-sectional characteristics, and odor. This analysis clarified the similarities and key distinguishing features between *P. vietnamensis* and its related species (Figure 2), providing critical pharmacognostic identification criteria to support the further development and utilization of medicinal Panax resources.

### 2.2. Microscopic Identification Method

The microscopic identification method was a significant innovation in medicinal material identification. It gradually emerged in the 1970s and 1980s. Microscopic identification has been adopted by pharmacopeias in major countries. For example, in the United States Pharmacopeia/National Formulary (USP36-NF31), nearly 200 dietary supplement varieties include microscopic identification items for each plant-based material and powder. The 8th edition of the European Pharmacopoeia (Ph.Eur.) includes 269 monographs for plant drugs and their preparations, with each plant drug’s identification section containing microscopic identification, excluding those without tissue characteristics, such as plant oils and extracts. The 16th revision of the Japanese Pharmacopoeia (JP) contains 276 entries for medicinal herbs, with a significant number of herbs, powdered medicinal materials, and powders used in formulated medicines, including concise microscopic identification items. In 1977, it was first included as a microscopic identification item in the *Chinese Pharmacopoeia* [43]. The microscopic morphological features of internal structural tissues and cells, along with their contents, in organisms such as plants, animals, and microorganisms, exhibit stability under specific environmental conditions and demonstrate specificity within a certain range. This forms the theoretical basis for the microscopic identification of traditional Chinese medicines [44]. Since the macroscopic identification of herbal materials is often subjective, potential substitutes or adulterants may appear highly similar to the genuine species, making microscopic identification a critical complementary method. The scanning electron microscope (SEM) plays a significant role in this context [45]. In herbal material identification, SEM provides highly detailed structural information, enhancing the accuracy of species differentiation. For example, Gou et al. [46] used an electron microscope to observe significant differences in the dorsal leaves of six types of honeysuckle, thus enabling species differentiation. Yan et al. [47] employed a phloroglucinol staining method to observe cross-sections of the stems of *Dendrobium officinale*, finding that its epidermal cells were easily stained due to their high degree of lignification. In contrast, epidermal cells of other *Dendrobium* species exhibited notable differences in lignification, shape, and number of layers, enabling rapid differentiation. In addition, Calcofluor White is used for staining cellulose and is suitable for fluorescence microscopy to observe its distribution. It can be used to differentiate cellulose content and distribution variations in plant cell walls among different species. Toluidine Blue O stains lignin and pectin and is suitable for optical microscopy to observe their distribution, aiding in species identification [48]. Solophenyl Flavine 7GFE is used to stain xyloglucans and is suitable for fluorescence microscopy to visualize their distribution. It helps differentiate xyloglucan content and distribution among plant species, assisting in species classification. Basic Fuchsin stains lignin and is suitable for fluorescence microscopy to analyze its distribution. It can be used to distinguish lignin content and distribution across different plant species, contributing to species identification [49]. Calcium oxalate crystals, as secondary metabolites in the growth and development of plants, are widely present in the tissue cells of medicinal plants. In microscopic identification, calcium oxalate crystals are characterized by their stability and distinct morphology, making them a key indicator for the identification of traditional Chinese medicines. Liu et al. [50] identified the presence or absence of calcium oxalate crystals, finding that the parenchyma cells of *Panax notoginseng* contained clusters of calcium oxalate, whereas counterfeit samples did not, allowing for accurate identification. The polarizing microscope, also known as a polarizing light microscope, is an optical microscope with an added polarization device. Polarized light shows a dark field when passing through isotropic materials, while anisotropic samples reveal distinct interference patterns. Zhao et al. [51] used the polarizing microscope to observe starch granules in *Astragalus membranaceus*, calcium oxalate crystals in *Panax ginseng*, stone cells in *Prunus persica*, and vessels in *Cornus officinalis*. However, this method relies heavily on the skills and experience of specialized personnel.

### 2.3. Physicochemical Identification Method

In the late 1980s, with the advancement of scientific methods, physicochemical identification methods gradually emerged. This marked the transition of medicinal material identification from traditional experience to scientific analysis, laying the foundation for the modernization of medicinal material research and application. Traditional Chinese medicine (TCM) physicochemical identification involves the use of physical, chemical, or instrumental analytical methods to verify the authenticity, purity, and quality of medicinal materials. Physicochemical identification analyzes the presence and concentration of key chemical constituents or active ingredients in medicinal materials, as well as the presence of harmful substances. The experimental methods for physicochemical identification of TCM typically involve using small quantities of crude powdered herbs, thin sections, extracts, or preliminarily isolated components for qualitative and quantitative analysis [52]. A color reaction, also known as a chromogenic reaction, occurs when a reagent is added to a medicinal material, resulting in a color change. Zhan et al. [53] employed reduction color reactions, complexation reactions with metal salt reagents, alkaline reactions, pentachloro-antimony reactions, and the Gibbs reaction to identify flavonoids, the active constituents of *Epimedium*. Among these, the alkaline color reaction was found to be the simplest and most convenient. Zhang et al. [54] utilized color reactions for the qualitative analysis of flavonoids in Mongolian medicine, Agi crude drugs, and carbonized drugs. Rutin was used as a reference compound, and the total flavonoid content was determined using ultraviolet spectrophotometry. Precipitation reactions refer to specific chemical reactions in which certain constituents of medicinal materials react with specific reagents to form precipitates for identification purposes. Yu [55] employed the Soxhlet extraction method, extracting fenugreek with 85% ethanol, adjusting the pH with hydrochloric acid, and allowing overnight precipitation. The supernatant was then loaded onto an AB-8 macro-porous resin and subjected to gradient elution using ethanol solutions of 10%, 30%, 50%, 70%, and 90%. Only the 70% and 90% ethanol fractions exhibited positive reactions, indicating that alkaloids were present in these eluates. For medicinal materials suitable for fluorescence analysis, some inherently exhibit fluorescence under visible or ultraviolet light, which can be used for qualitative identification. The solvent medium can be either aqueous or organic. In contrast, some medicinal materials do not exhibit intrinsic fluorescence, necessitating chemical modification through fluorescence derivatization for broader analytical application. For example, thin sections of *Ophiopogon japonicus* observed under ultraviolet light (365 nm) exhibit a pale blue fluorescence. In practice, most medicinal materials have complex chemical compositions, which may cause interference or lack intrinsic fluorescence. Acid, alkali, or organic solvent treatments are often required to extract fluorescent compounds [56]. For instance, crude powder of *Zanthoxylum bungeanum* is soaked in chloroform, and the filtrate is spotted onto filter paper and dried. Under ultraviolet light (254 nm), it exhibits a scarlet fluorescence. Similarly, *Hippophae rhamnoides* powder extracted with ethanol and observed under ultraviolet light (365 nm) emits a yellow–green fluorescence [57]. Ding [58] applied fluorescence analysis to distinguish *Chaenomeles speciosa* from its adulterants, where the genuine species exhibited blue fluorescence, whereas Tibetan Chaenomeles showed green fluorescence, demonstrating significant differences useful for authentication.

### 2.4. Microcharacteristic Identification Method

**Figure 3:**
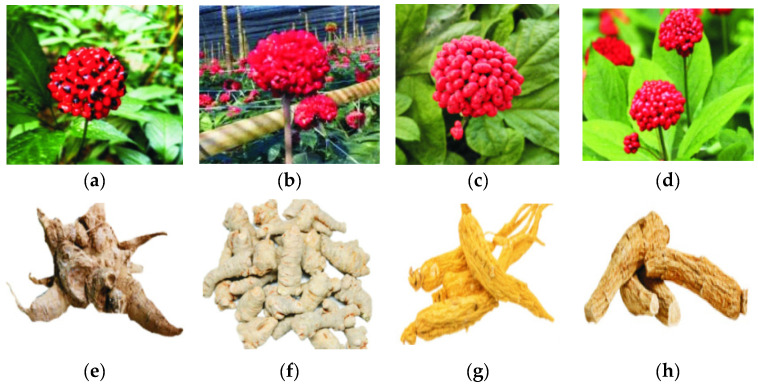
Vietnamese ginseng, Notoginseng, Ginseng, and American ginseng, including their raw plants and dried products. (**a**) Vietnamese ginseng. (**b**) Notoginseng. (**c**) Ginseng. (**d**) American ginseng. (**e**) Vietnamese ginseng. (**f**) Notoginseng. (**g**) Ginseng. (**h**) American ginseng.

**Figure 4:**
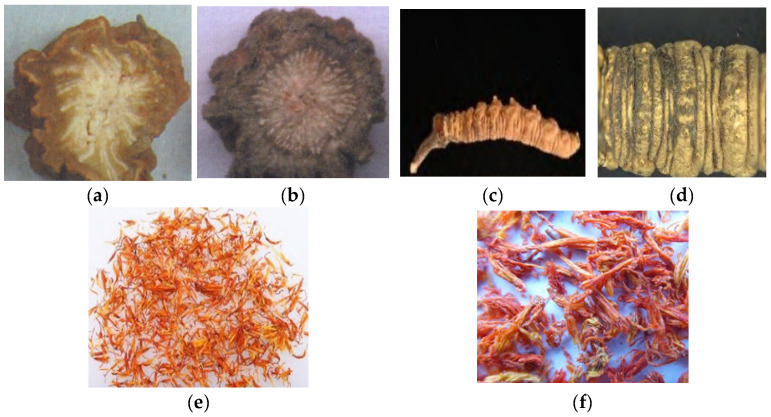
(**a**) Genuine Dan Shen cross-section; (**b**) counterfeit *Burdock* cross-section; (**c**) macroscopic morphology of *Cordyceps sinensis*; (**d**) micro-morphology of *Cordyceps sinensis*; (**e**) normal safflower; (**f**) safflower after extraction and staining with increased weight [62] (Reprinted with permission from Ref. [62]. Copyright 2024, Shanxi College of Pharmaceutical Sciences).

Traditional trait identification provides crucial support for the quality evaluation of traditional Chinese medicines (TCMs) and is often able to achieve quick on-site identification in practical applications. However, it cannot identify subtle features on the surface of TCM materials that are not easily visible to the naked eye and, to some extent, no longer meets the demands of current work. The micro-trait identification method for TCM materials utilizes instruments to observe minute traits on the surface (including cross-sections) of herbal materials and TCM slices, which are not visible to the naked eye, using these observations as a basis for identification [59]. As mentioned above, microscopic identification focuses more on observing the internal microscopic structures of the materials, especially for identifying broken or processed samples. The stereomicroscope, with a magnification range of 5–100 times, does not penetrate the object but provides better observation of the surface texture, irregularities, patterns, and color of the material. It directly reveals the original color and shape of the raw material and allows the observation of many features that traditional trait identification cannot detect and that microscopic identification may fail to resolve [60]. Ma et al. [61] used a stereomicroscope and scanner to analyze the external shape, surface, and cross-sectional features of *Salvia miltiorrhiza* and *Arctium lappa* roots. They found that the cross-section of *Salvia miltiorrhiza* exhibited sparse, radially distributed yellowish-white vascular bundles with indistinct vascular cambium rings, while *Arctium lappa* showed densely arranged, regular vascular bundles and prominent cambium rings, providing a reference for authenticity identification as shown in Figure 3a,b. Yang et al. [62] conducted a study on *Cordyceps sinensis* and safflower using stereomicroscopy. Due to the growth process of *Cordyceps sinensis*, where its stalk grows in the soil, it is difficult to completely remove the attached dirt and other impurities during processing. Even though it may appear clean to the naked eye, large amounts of soil can still be seen under the stereomicroscope. If not properly handled, consuming *Cordyceps sinensis* containing significant amounts of soil could affect its therapeutic efficacy, as shown in Figure 4c,d. Safflower, a commonly used medicinal herb, is often adulterated. The primary adulterant used for weight enhancement is magnesium sulfate, which has a laxative effect and can cause side effects when consumed. There are various methods of adulteration, including adding natural colorants or coloring after extraction, with the latter being more common. The adulterated safflower closely resembles the authentic product in appearance and is typically mixed in appropriate proportions without affecting the standard pharmacopoeia requirements. However, the quality control of safflower is challenging using only physical and chemical tests, making it difficult to detect adulteration. The application of micro-trait identification can effectively control the quality of safflower. Normal safflower has red flower petals and yellow stamens, with only a small amount of pollen attached to the surface. In contrast, the adulterated safflower appears wrinkled with uneven color on its surface. The yellow parts may have red spots or be entirely red, and white granular particles are visible on the surface, as shown in Figure 4e,f.

Traditional identification methods evaluate the authenticity and quality of herbal medicines by observing and analyzing their external characteristics and internal tissue structures. These methods are advantageous due to their simplicity and low cost. However, they are highly dependent on the knowledge and experience of the practitioners, resulting in a certain degree of subjectivity. Additionally, traditional methods are insufficient to meet the demands of large-scale, high-throughput identification and cannot effectively analyze the chemical composition of the materials. To address these limitations, the application of modern scientific methods provides more efficient, accurate, and reliable solutions for the identification of herbal medicines.

## 3. Modern Identification Methods for Food-Medicine Homologous Herbal Materials

With the advancement of identification methods for medicinal materials of Food-Medicine homologous origin, biomimetic recognition, chemical analysis, spectroscopic analysis coupled with chemometrics, and molecular biological identification have progressively become the primary modern methods for both qualitative and quantitative analysis of these materials. Biomimetic recognition methods primarily include electronic noses, electronic tongues, and visual biomimetic recognition, all of which simulate biological sensory functions for efficient identification of medicinal material characteristics. Chemical analysis methods include chromatography, mass spectrometry, and their combined techniques, focusing on the precise separation and quantitative analysis of chemical components. Spectroscopic analysis coupled with chemometrics relies on characteristic spectral measurements to study the structure or chemical composition of compounds [63], offering advantages, such as ease of operation and no complex sample preparation [64]. Among molecular biological identification methods, DNA barcoding is the most representative technique [65], enabling rapid and accurate species identification by analyzing specific gene sequences. This has facilitated the transition of medicinal material identification from traditional morphological analysis to molecular-level techniques [66]. In recent years, the application of Artificial Intelligence (AI) algorithms, particularly Machine Learning (ML) and Deep Learning (DL), has significantly advanced the field of Traditional Chinese Medicine (TCM) identification. These technologies provide efficient, accurate, and automated solutions for identifying, classifying, and quality-controlling TCM materials. The integration of AI algorithms in TCM identification techniques is transforming the traditional methods, improving precision, and enhancing the speed of analysis.

### 3.1. Chemical Analysis Methods

#### 3.1.1. Biomimetic Recognition Method

**Figure 5:**
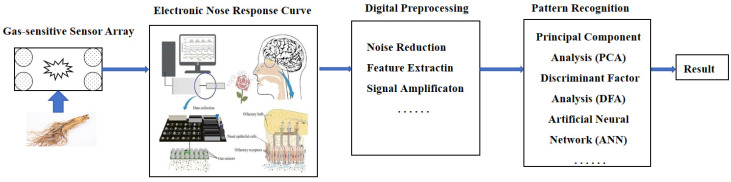
Information processing flow of the electronic nose.

Olfactory biomimetic technology is a product of research on animal olfactory systems, combined with sensor technology, electronics, and computer technology. It mimics the working model of olfactory epithelial cells in the human brain to detect odors. The electronic nose consists of an array of chemical sensors enclosed in a sealed container. Target mixtures enter the container in gaseous form, and the reaction between the gas and sensors causes changes in the electrical conductivity of the sensors. Each sensor responds uniquely to certain components. Within the array, each sensor has different characteristics (such as coating, operating temperature, etc.). Therefore, the varying electrical responses (i.e., voltage outputs) of the sensors in the array combine to form a fingerprint (or pattern) that is unique to a specific odor. By using pattern recognition algorithms on the fingerprint, different odor components can be identified [67], as illustrated in Figure 5. Significant differences in the characteristic odors of medicinal materials from various regions or varieties, along with large price disparities, often lead to issues such as counterfeit substitution and mixed varieties. Wu et al. [68] developed an electronic nose system to collect odor data from six types of *Sichuan pepper* samples, which were classified using a support vector machine (SVM) model, achieving an accuracy rate of 94.27%. Gan et al. [69] utilized an electronic nose to distinguish the varieties and origins of *Atractylodes lancea*. By rapidly detecting volatile aromatic compounds, the electronic nose revealed that the primary aroma characteristics of *Atractylodes lancea* include spiciness, sweetness, and fruity notes. Significant differences in sweetness and spiciness were observed among samples from different regions. This study provides a rapid and accurate strategy for quality control and market management of *Atractylodes lancea*. Moreover, it offers a novel method for quality evaluation and origin identification of other medicinal materials.

Visual bionics is a rapidly evolving electronic sensing technology that can accurately describe the color, size, and surface structure of medicinal plants by capturing, processing, and analyzing images [70]. Proper lighting plays a critical role in obtaining high-quality images, as a good light source can effectively reduce interference factors, such as reflections, shadows, and noise, thereby minimizing the workload of image processing [71]. Li et al. [72] utilized the IRIS VA400 electronic eye device for color calibration of white and sample materials under top lighting conditions using a 24-color board. They performed a quantitative analysis of visual features such as color and texture. For each sample, a random area of approximately 10 × 10 cm^2^ was selected for photography, with a 5 mm aperture and simultaneous activation of both upper and lower backlighting to eliminate background interference. Three images were taken for each sample to capture visual information from different angles. The Partial Least Squares Discriminant Analysis (PLS–DA) model constructed, based on electronic eye data performed well in distinguishing white materials from other decoction pieces, with an accuracy of 98% for the calibration set and 97.06% for the validation set. The Support Vector Machine (SVM) model achieved an accuracy of 99% for the calibration set and 97.06% for the validation set. The Backpropagation Neural Network (BP–NN) model showed an accuracy of 99% for the calibration set and 97.06% for the validation set. Cui et al. [73] employed an electronic nose, electronic tongue, electronic eye, and near-infrared spectroscopy (NIR) combined with data fusion techniques for authenticity identification and species identification of *Fritillariae cirrhosae* and its counterfeit products. Using machine learning algorithms such as Partial Least Squares Discriminant Analysis (PLS–DA) and Principal Component Analysis Discriminant Analysis (PCA–DA), the researchers constructed identification models based on both single-source data and multi-source data fusion. The results showed that the electronic eye and near-infrared spectroscopy performed best in authenticity identification and species identification of *Fritillariae cirrhosae*, with an accuracy of 97.50%, respectively. By applying data fusion techniques, the four-source data fusion model further improved the accuracy of authenticity identification to 98.75%, and the accuracy for species identification reached 97.50%. This study provides an efficient and accurate method based on artificial intelligence and data fusion for quality evaluation of Chinese medicinal materials, offering new technical solutions for quality control and market management of traditional Chinese medicine. However, visual bionic systems have certain limitations, as they can only obtain chemical composition information of medicinal plants within specific spectral wavelengths, making it challenging to acquire additional information [74].

Taste bionic technology mimics the working model of human taste cells and receptor sensing to detect the “flavor” of liquids. The electronic tongue uses biomimetic materials as sensitive membranes for its sensors. When the lipid-like membrane comes into contact with taste substances on one side, the membrane potential changes, producing a response. Each independent sensor in the sensor array acts like a taste bud on the tongue, with each sensor detecting a specific group of chemical substances [75]. Different chemical substances are detected, and the various signals are collected and input into a computer. The computer replaces the brain’s function in biological systems, analyzing and processing the data through software to differentiate and identify the substances, ultimately providing sensory information for each substance. These taste sensors in the sensor array are characterized by high sensitivity, reliability, and repeatability, and they can also measure the concentration of certain components [76]. Yan et al. [77] conducted a fusion analysis of electronic nose and electronic tongue data from heated black goji berry extracts, integrating this with linear discriminant analysis (LDA), and achieved an overall prediction accuracy of 92.6%, which was significantly higher than using either the electronic nose or electronic tongue alone. This approach successfully distinguished black goji berries from different regions. For instance, Li et al. [78] utilized Artificial Intelligence Sensing (AIS) and Multi-Source Information Fusion (MIF) technologies to conduct rapid quality identification of different grades and adulteration levels of *Panax notoginseng* powder (PNP). In their study, sensory information from samples was collected using an electronic tongue (ET), electronic nose (EN), and electronic eye (EE), combined with saponin content measured by High-Performance Liquid Chromatography (HPLC). Qualitative and quantitative models were constructed based on these data. The results demonstrated that the model based on MIF technology achieved 100% accuracy in identifying grades and detecting adulteration of PNP. For predicting adulteration ratios and total saponin content, the model’s coefficient of determination (R^2^) exceeded 0.99, and the root mean square error (RMSE) was as low as 0.0109 and 0.0123, respectively. This indicates that the method can effectively and rapidly assess the quality of PNP, providing a novel technological approach for quality control of powdered foods or pharmaceuticals.

#### 3.1.2. Chromatographic Methods

**Figure 6:**
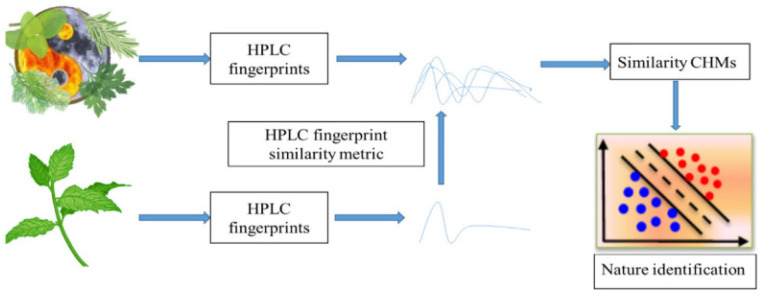
Cold–hot nature identification model based on HPLC similarity metric. The red and blue dots represent hot-natured and cold-natured traditional Chinese medicine (TCM) samples, respectively. They serve as reference samples in the model to identify the cold or hot nature of unknown TCM samples based on HPLC fingerprint similarity [80].

Chromatographic identification methods are extensively employed for the separation, identification, and quantitative analysis of mixture components, offering advantages such as robust separation capacity, broad applicability, and exceptional qualitative and quantitative precision. This approach is based on differences in distribution coefficients or adsorption capacities of mixture components between the stationary and mobile phases, enabling effective separation and analysis. Thin Layer Chromatography (TLC) is the earliest chromatographic method extensively applied to the identification of Chinese medicinal materials, whereas High-Performance Liquid Chromatography (HPLC) has emerged as a principal technique for this purpose. The theory of medicinal properties is a core concept in traditional Chinese medicine (TCM). The cold and hot properties of medicines are integral to TCM constitution theory. Hot-natured medicines are used to treat cold syndromes, while cold-natured medicines are used to treat hot syndromes. Fu et al. [79] employed bioinformatics approaches to explore the cold and hot properties of Chinese medicinal materials. Their findings indicated that cold-natured medicines tend to affect cell growth and proliferation, exhibit sedative functions, and are associated with diseases classified under “mental and behavioral disorders”. In contrast, hot-natured medicines are linked to inflammation and immune modulation, exhibit cardioprotective effects, and are associated with diseases categorized as “endocrine, nutritional, and metabolic disorders”. Thus, accurately understanding the nature of Chinese medicines is critical for advancing TCM research. Wei et al. [80] used High-Performance Liquid Chromatography (HPLC) to separate the chemical components of Chinese herbal medicines. A total of 61 medicinal materials with well-defined cold or hot properties were selected as research subjects, leading to the development of a Cold–Hot Nature Identification Scheme (CHNIS) and a model for evaluating the cold–hot properties of Chinese medicines (as illustrated in Figure 6). For a Chinese herbal medicine (CHM) with unknown cold or hot properties, the first step involves extracting its chemical composition using an HPLC fingerprint. Subsequently, the Mahalanobis distance is learned to calculate the similarity between the HPLC fingerprint of the query CHM and CHMs with known properties. The learned Mahalanobis distances are then arranged in ascending order based on their metric values. The HPLC fingerprint with the smallest distance is selected to identify the most similar CHM. The results indicated an overall identification accuracy of 80.3%, validating the hypothesis that Chinese medicines sharing similar cold–hot properties demonstrate significant similarity in their material composition. Furthermore, chromatographic techniques can be integrated with a variety of detectors. Tian et al. [81] employed a combination of High-Performance Liquid Chromatography–Evaporative Light Scattering Detector (HPLC–ELSD) and High-Performance Thin-Layer Chromatography (HPTLC) to analyze the primary bioactive components of *Bupleurum*, i.e., Bupleurum saponins. The study evaluated the quality of 33 authenticated *Bupleurum* samples and 31 commercially sourced *Bupleurum* samples. Similarly, Chen et al. [82] devised an HPLC method integrated with a Photodiode Array Detector (PDA) to simultaneously determine norepinephrine and dopamine in extracts from various parts of *Purslane*. The results showed that the calibration curve for the analytes exhibited a correlation coefficient exceeding 0.999, confirming the method’s exceptional sensitivity and accuracy.

#### 3.1.3. Mass Spectrometry

Mass spectrometry (MS) involves converting molecules or atoms in a solution into charged ions. These ions are subsequently separated by a mass analyzer based on their mass-to-charge ratios. The resulting data are recorded by a detector, producing a mass spectrum [83]. Compared to chromatographic methods, mass spectrometry provides significantly faster detection speeds. Since most MFH plants are natural products, variations in weather and soil conditions, the environmental quality of the cultivation areas, as well as processing techniques for these plants as pharmaceutical formulations and their storage and transportation may affect the extent of toxic element contamination. According to the 2010 edition of the Pharmacopoeia of the People’s Republic of China, the exceedance rates for arsenic (As), cadmium (Cd), mercury (Hg), and lead (Pb) were 14, 16, 6, and 18%, respectively. Based on the limits set by the Chinese National Standard (CNS) GB 2762-2012 [84], the exceedance rates for As, Cd, Hg, and Pb were 44, 20, 12, and 34%, respectively. Therefore, special attention should be paid to the potential risks of toxic element contamination in MFH plants. Fu et al. [85] devised an analytical method utilizing Inductively Coupled Plasma Tandem Mass Spectrometry (ICP–MS/MS) to precisely quantify four toxic elements (As, Cd, Hg, and Pb) in 50 commonly used medicinal and edible plants from China, facilitating safety assessments and regulatory management of these elements. Additionally, Direct Analysis in Real Time Mass Spectrometry (DART–MS) is an innovative rapid identification technique. It has been successfully applied to analyze active ingredients in traditional Chinese medicine (TCM). This method eliminates the need for complex sample preparation, enabling samples to be directly analyzed and ionized in open air within seconds. It resolves the time-consuming and solvent-intensive limitations of chromatographic techniques, significantly reducing analysis time. However, certain compounds that are challenging to ionize may require additional derivatization steps. Moreover, as mass spectrometry generally necessitates ionization in a closed environment, sample pretreatment and chromatographic separation are typically required prior to the analysis of TCHM. Ginseng, Coptis Rhizome, and Scutellaria Root are traditional Chinese medicinal materials that also possess homologous Food-Medicine properties. Ginseng is renowned for its primary active components, ginsenosides, which are widely consumed not only for their tonic effects but also for their use in traditional Chinese medicine to treat various diseases. The main component of Coptis Rhizome, berberine, exhibits antibacterial and anti-inflammatory pharmacological effects. In addition to its medicinal applications, it is also incorporated into certain functional foods for its health benefits. Scutellaria Root contains flavonoids, such as baicalein and wogonin, which have antioxidant and anti-inflammatory properties, making it widely utilized in both traditional medicine and functional food products. Wang et al. [86] utilized the DART–MS technique to analyze eight traditional Chinese medicines (TCMs), including Scutellaria Root, Coptis Rhizome, and Ginseng. DART-MS is an ambient ionization technique that allows for direct sampling and ionization of the sample in open air, requiring little or no sample pretreatment. DART-MS generates ions directly from the sample surface by exposing the sample to an ionization gas stream, which reacts with atmospheric components, such as nitrogen, oxygen, water, etc. This method is rapid, environmentally friendly, and easy to use, making it suitable for the analysis of various samples. By optimizing the helium gas temperature, they successfully detected pseudo-ginsenoside F11, compound K, protopanaxatriol, and protopanaxadiol without the need for derivatization. Additionally, they explored the ionization mechanisms of these compounds. Future research should prioritize optimizing the DART ionization temperature and its performance across diverse environmental conditions to improve the detection sensitivity and stability of TCM components.

#### 3.1.4. Chromatography–Mass Spectrometry Coupling

**Figure 7:**
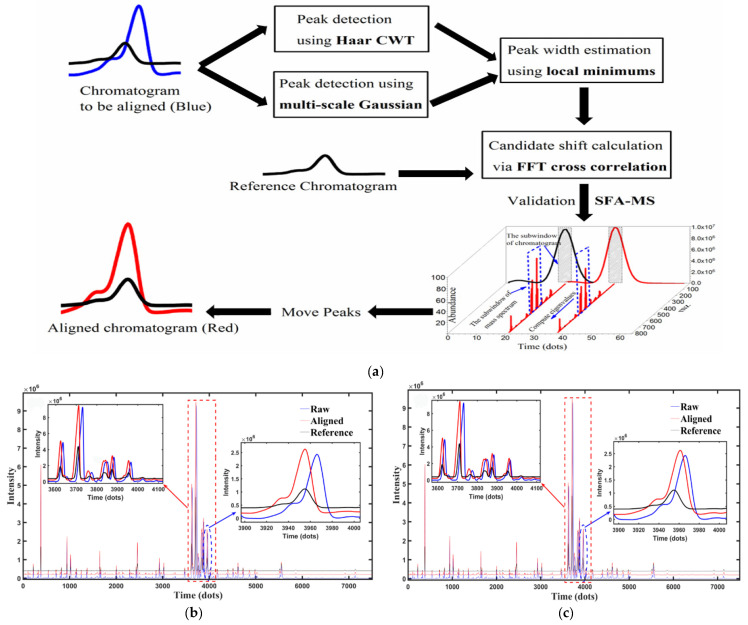
(**a**) Flow chart of an improved SFA–MS algorithm; (**b**) The alignment results by an improved SFA–MS algorithm; (**c**) The alignment results by CAMS algorithm [90].

Chromatography–mass spectrometry (Chromatography–MS) combines the separation power of chromatography for complex mixtures with the high sensitivity and resolution of mass spectrometry. It is among the most widely used analytical tools for identifying components in traditional Chinese medicine (TCM). Among these methods, High-Performance Liquid Chromatography–mass spectrometry (HPLC–MS) is particularly prevalent [87]. Wu et al. [88] reviewed the application of Liquid Chromatography–mass spectrometry (LC–MS) in the identification of medicinal and edible plants, emphasizing its exceptional sensitivity and selectivity in chemical composition identification and quality control. LC–MS has increasingly become a mainstream technique. Li et al. [89] successfully identified anticancer-active compounds in turmeric using a combination of countercurrent chromatography and ultra-high-performance liquid chromatography coupled with high-resolution mass spectrometry. They subsequently confirmed the significant anticancer activity of four compounds through cellular assays. He et al. [90] adopted a novel improved algorithm combining sub-window factor analysis with mass spectrometry information (SFA–MS) to align chromatograms and address the bias caused by chromatographic peak shifts. They differentiated Bupleurum samples from different geographical origins using hierarchical clustering analysis (HCA) and principal component analysis (PCA). Figure 7 illustrates the workflow of the improved SFA–MS algorithm and compares its results with those of CANS. The study demonstrated that the SFA–MS algorithm is more robust than the CAMS algorithm when handling overlapping peaks, significantly improving the differentiation of geographical origins of Bupleurum samples. This approach provides a scientific basis for ensuring the authenticity and quality control of medicinal materials. Jia et al. [91] developed an offline multidimensional chromatography–high-resolution mass spectrometry method to identify the chemical components in Ginseng, Notoginseng, and other medicinal herbs. They identified 1561 previously unknown ginsenosides, demonstrating the potential of chromatography–mass spectrometry coupling for discovering novel lead compounds in traditional Chinese medicine (TCM). Gikas et al. [92] employed ultra-high-performance liquid chromatography–high-resolution mass spectrometry, utilizing specific chromatographic columns and mobile phase conditions, and acquired mass spectrometry data using a mass spectrometer. They annotated the mass spectral features by referencing multiple databases and a self-assembled mass spectrometry library, revealing the chemical composition differences of saffron from various countries and providing key insights into saffron origin identification. Yoon et al. [93] explored the identification of ginsenosides in *American ginseng* (*P. quinquefolius* L.) harvested from regions within the Protected Designation of Origin (PDO) (the United States and Canada) as compared to those from non-PDO regions (Shandong Province and Northeast China). Through the comparison of retention time differences, a quantitative analysis was conducted on 11 major ginsenoside compounds (Rb1, Rb2, Rb3, Rc, Rd, Re, Rf, Rg1, Rg2, F2, and Rg3) in the roots of American ginseng cultivated in the United States, Canada, and China. The contents of Rg2, Rb2, Rd, Rg3, and F2 in the PDO samples were higher than those in the non-PDO samples. Chemometric models, such as Linear Discriminant Analysis (LDA) and Random Forest (RF), successfully differentiated American ginseng from four other sources using ginsenoside markers. Both models achieved a classification accuracy of over 90% in distinguishing PDO and non-PDO American ginseng samples, demonstrating the effectiveness of combining chemical composition with statistical modeling in identification studies.

The advantages and disadvantages of using chromatography to evaluate the quality of medicinal plants are undeniable. This technique requires expensive equipment and time, and the reagents used can also have an environmental impact. However, due to its powerful separation capability, chromatography has become the dominant method for quantitative analysis of medicinal plants. Liquid chromatography (LC), particularly high-performance liquid chromatography (HPLC) and ultra-high-performance liquid chromatography (UHPLC), are key tools for measuring the secondary metabolites of medicinal plants, thereby facilitating quality control [94]. The high precision and resolution of these techniques make them essential for the quantitative analysis and adulteration detection of medicinal plants [95]. To further characterize the chemical structures of complex mixtures, the continuous advancement of chromatographic equipment and the development of environmentally friendly solvents are inevitable trends [96]. Mass spectrometry (MS) is a conventional detection technique that, by providing MS^n^ information and a broad dynamic linear range, can elucidate and quantify secondary metabolites in medicinal plants on a large scale. MS^n^ (Multistage Tandem Mass Spectrometry) is a mass spectrometry technique in which “^n^” represents the number of mass spectrometry stages. In mass spectrometry analysis, MS^n^ technology provides more detailed structural information through multiple (n times) mass spectrometric analyses. Specifically, MS^n^ can progressively fragment and analyze the target compound, thereby improving the accuracy and reliability of identification. Primary mass spectrometry, used to detect the mass-to-charge ratio (*m*/*z*) of molecular ions, providing molecular weight information. Secondary mass spectrometry, in which molecular ions are further fragmented using collision-induced dissociation (CID) or other techniques to generate fragment ions, providing structural information. In multistage mass spectrometry, multiple fragmentation and detection steps are performed to achieve deeper structural analysis. However, its high cost, complex operation, labor-intensive sample preparation, interference effects, and issues with quantitative accuracy still limit its widespread application in certain fields. When using mass spectrometry, it is essential to consider factors such as the sample characteristics, research objectives, and equipment availability to determine whether mass spectrometry should be used or whether other analytical methods should be combined to address its limitations. The combination of chromatography and mass spectrometry offers a feasible scientific solution for the quality evaluation of both medicinal and edible plants. This integrated technique enhances analytical capabilities through coupling, ensuring high accuracy and precision [97]. However, its high equipment requirements, operational complexity, and sample processing demands also limit its widespread adoption and application.

### 3.2. Spectroscopic Methods

#### 3.2.1. Near-Infrared Spectroscopy

**Figure 8:**
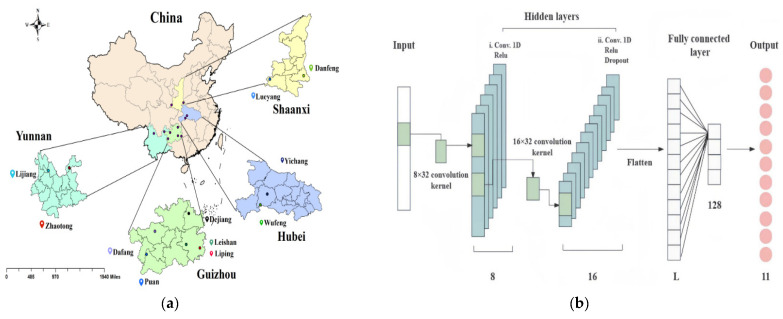
(**a**) Geographical Origins of Gastrodia elata; (**b**) The Framework of the 1D-CNN Model. (Reprinted with permission from Ref. [103]. Copyright 2023, MDPI [102]).

Near-Infrared Spectroscopy (NIR), located between the visible and mid-infrared spectra, is a simple, rapid, accurate, and non-destructive analytical method that has been widely applied in process analysis and quality control in recent years [98,99]. NIR offers significant penetration ability and requires minimal sample preparation, making it ideal for directly detecting solids, powders, and liquids. The technique primarily records spectral bands associated with the molecular vibrations of hydrogen-bonded groups, such as C-H, N-H, and O-H, providing characteristic information about hydrogen-containing groups in compounds [100]. Arslan et al. [101] employed Fourier Transform–Near-Infrared Spectroscopy (FT–NIR) in conjunction with a Genetic Algorithm–Partial Least Squares (GA-PLS) model for the quantitative analysis of flavonoid components in black goji berries. The results indicated that the RPD value of the model exceeded 2.0, demonstrating good stability and predictive capability. Chen et al. [102] analyzed 250 *Panax notoginseng* samples from various regions of China, including Yunnan, Tibet, Guangxi, and Guizhou, using Near-Infrared Spectroscopy (NIR). The raw spectral data were preprocessed using Standard Normal Variate (SNV) transformation and the first derivative, then a discriminant model was developed using Partial Least Squares Discriminant Analysis (PLSDA). The study successfully identified the geographic origin of the samples, providing a scientific basis for the traceability of medicinal material origins. Ma et al. [103] combined Visible–Near Infrared Spectroscopy (Vis–NIR) with a one-dimensional Convolutional Neural Network (1D-CNN) model (as shown in Figure 8) to analyze the geographical origins and physiological activity component contents of *Gastrodia elata*. The structure of a 1D-CNN is similar to that of a traditional CNN, consisting of an input layer, convolutional layers, grouping layers, pooling layers, fully connected layers, and dense layers, which are used for feature extraction, learning, and providing numerical outputs for classification or regression tasks [104,105]. However, it is more powerful than traditional CNNs in terms of model representation. The convolutional layers are composed of multiple convolutional kernels. Convolution with these kernels on raw data is considered for the extraction of features that are represented by the convolutional kernels. The number of kernels determines the number of features generated. Different activation functions are used to display complex features. Eight kernels are used in the first convolutional layer to capture low-level local features. The second convolutional layer uses 16 kernels to further aggregate and abstract these features, capturing higher-level features. This hierarchical feature extraction process helps the model better understand the structure of the data. The results indicated that this method could accurately distinguish 11 geographic origins of Gastrodia elata and predict the contents of eight active components. The model achieved an F1 score of 1.0000, with RMSEP values ranging from 0.2881 to 1.2965 and R^2^ values between 0.9094 and 0.9323, demonstrating the high efficiency and accuracy of the 1D-CNN model. Kar et al. [106] employed near-infrared (NIR) spectroscopy to quantitatively analyze the presence of 1–25% Sudan I in turmeric powder samples, developing two regression models: principal component regression (PCR) and partial least squares regression (PLSR). The results indicated that, based on the limit of detection (LOD), the lowest detectable concentration using NIR spectroscopy and the two regression models was approximately 0.3%, with PLSR demonstrating superior accuracy compared to PCR.

#### 3.2.2. Hyperspectral Imaging

**Figure 9:**
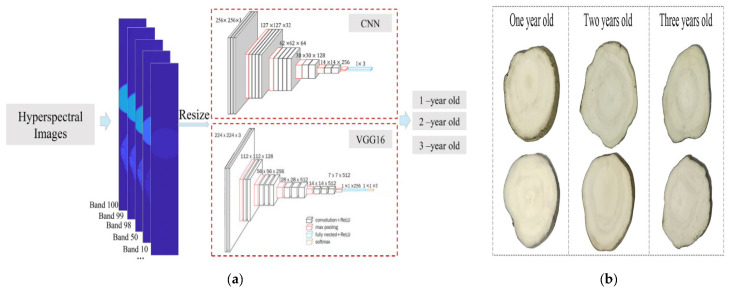
(**a**) Convolutional Neural Network and VGG16 Framework; (**b**) cross-sections of Pueraria lobata samples from different years [111].

Hyperspectral imaging (HSI) is a non-contact detection method that integrates imaging with spectral analysis. This method captures detailed spectral information of traditional Chinese medicinal herbs and combines it with spatial data to generate hyperspectral images that encompass both spatial and spectral characteristics. Currently, HSI is extensively applied in fields such as food [107], agriculture [108], and herbal medicine [109] analysis. For example, Zhang et al. [110] employed near-infrared hyperspectral imaging in conjunction with deep learning techniques to determine the total flavonoid content in the dried fruits of *Lycium ruthenicum*. The study demonstrated that this method provides predictive performance for total anthocyanins, flavonoids, and phenolic compounds in *Lycium ruthenicum* that is comparable to traditional methods. Furthermore, in another study, Zhang et al. [111] combined a convolutional neural network classification framework with HSI to capture hyperspectral images of the external surface and cross-sections of *Pueraria lobata* at different ages, as shown in Figure 9. The CNN architecture consists of four convolutional layers, with batch size, training epochs, and learning rate set to 4, 100, and 0.003, respectively. In addition to the CNN-based network, we also used WAVGG16 in this study to identify the growth year of PTR. VGG16 is a specific convolutional neural network model with a total of 16 layers, including 13 convolutional layers and 3 fully connected layers. Compared to other network models, VGG16 employs a uniform 3 × 3 convolutional kernel throughout the entire process. This relatively small kernel size facilitates an increase in the depth of the network structure, while the sufficiently large number of parameters can be used to learn more complex patterns, leading to better classification performance. The results indicated that the F1 score of the model exceeded 90%, confirming the feasibility of using deep learning algorithms combined with HSI to identify the growth years of *Pueraria lobata*. These examples underscore the vast potential of combining hyperspectral imaging with deep learning methods for the quality detection and identification of traditional Chinese medicinal herbs. Vermaak et al. [112] explored the use of hyperspectral imaging (HSI) technology to distinguish adulterated or substituted Japanese star anise (*Illicium anisatum*) from Chinese star anise (*Illicium verum*) in dried products. The study utilized a hyperspectral imaging system with a broad spectral range (920–2514 nm) to capture images, employing principal component analysis (PCA) for data dimensionality reduction and partial least squares regression (PLSR) as the classification model. The results demonstrated that hyperspectral imaging technology can be successfully applied to identify whole dried fruits of *I. anisatum* and *I. verum*.

#### 3.2.3. Terahertz Spectroscopy

Terahertz (THz) radiation lies between microwave and infrared frequencies, ranging from approximately 0.1 to 10 THz. The characteristic THz spectrum can effectively identify biological molecules [113], cancer cells and drugs [114,115,116], crops [117,118], and gaseous components [119]. Recent theoretical and experimental studies have shown that the low-frequency vibrational and rotational modes of chemical components and secondary metabolites in traditional Chinese medicinal herbs primarily fall within the THz range. Therefore, Terahertz–Time-Domain Spectroscopy (THz–TDS), with its rapid, safe, and non-destructive advantages [120], has emerged as a promising tool for identifying medicinal herbs, garnering increasing attention.

**Figure 10:**
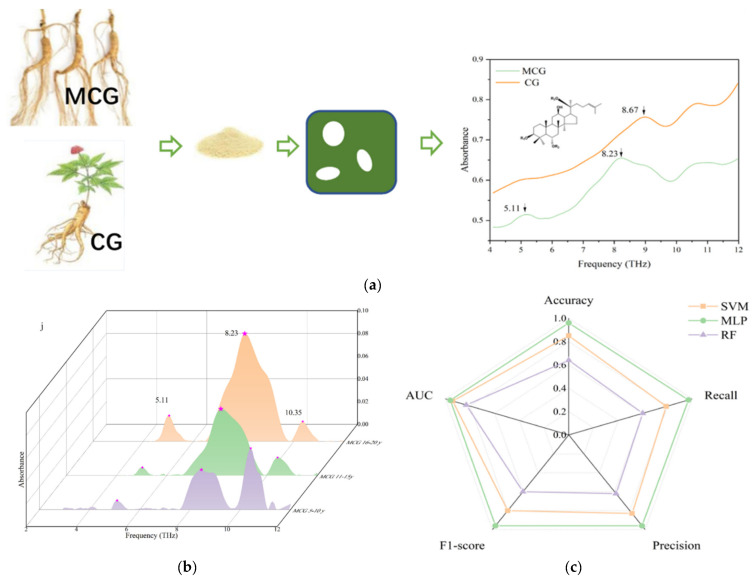
(**a**) Procedure for identifying MCG using terahertz fingerprint spectroscopy; (**b**) THz absolute spectral comparation of 3 MCG groups; (**c**) Metrics to evaluate the performance of classification models of RF, SVM and MLP. (Reprinted with permission from Ref. [122]. Copyright 2023, Elsevier Inc.).

Distinguishing authentic from counterfeit medicinal herbs is a prevalent issue in the market. For example, ginseng and American ginseng are often confused due to commercial interests or their similar appearance. To address this problem, Du et al. [121] employed terahertz spectroscopy in conjunction with variable importance analysis and data preprocessing methods, significantly enhancing the model’s discriminative power. The results demonstrated that the Orthogonal Partial Least Squares–Discriminant Analysis (OPLS–DA) model could successfully differentiate between *Fritillaria* samples and their counterfeit counterparts, achieving an accuracy rate of 100%. Additionally, Zhang et al. [122] utilized terahertz spectroscopy to differentiate high-altitude cultivated ginseng (MCG) from ordinary cultivated ginseng (CG), effectively distinguishing MCG from CG based on specific absorption peaks in the raw data combined with the OPLS–DA method. The predictive value Q2 at the intersection with the Y-axis was −1.32, and the coefficient of determination (R2) was 97.5%, indicating that the model was not overfitted and exhibited high explanatory and predictive power. The identification process is shown in Figure 10a. Furthermore, MCG with a growth period of 5–20 years was divided into three distinct groups based on the extracted terahertz spectral features, as shown in Figure 10b. Finally, three models—RF, SVM, and MLP—were constructed to train and predict the three growth age ranges of ginseng, with the prediction results presented in Figure 10c. It can be observed that MLP is the optimal algorithm for distinguishing MCG age, with an accuracy rate of 96.0%. This suggests that the method of classifying ginseng into three age groups using terahertz spectral signals and machine learning algorithms is a novel and promising approach, as it enables high precision and efficiency. However, this method also has some limitations. In the future, more ginseng samples can be collected to improve generalizability, and the total amount table can be optimized to achieve more accurate grouping. Furthermore, the quality and efficacy of medicinal herbs are influenced by storage conditions and geographical origin and can vary over time and with different growing environments. Li et al. [123] employed terahertz spectroscopy in conjunction with deep learning algorithms to effectively differentiate between *Chenpi* samples from various years and regions.

Pharmacological processing is a critical step in preparing traditional Chinese medicinal materials, significantly affecting their chemical composition and pharmacological activity. He et al. [124] employed terahertz (THz) spectroscopy to determine the absorption coefficients of raw and processed *Astragalus membranaceus*, using these values as feature data for hierarchical clustering analysis, achieving an accuracy rate of 94.4%. Furthermore, due to the diverse biological activities of flavonoids in traditional Chinese medicine, increasing attention has been given to this group of compounds. Yin et al. [125] employed terahertz–time-domain spectroscopy (THz–TDS) combined with chemometric methods to perform qualitative and quantitative analyses of structurally similar flavonoids within the 0.2–2.5 THz range. The study revealed that different flavonoids exhibited unique characteristic absorption peaks across distinct frequency bands, further validating the capability of the “terahertz fingerprint spectrum” to effectively distinguish between compounds. Similarly, Yan et al. [126] demonstrated that glycyrrhizic acid, glycyrrhetinic acid, and glycyrrhizin exhibit pronounced absorption features within the 0.3–1.72 THz range, offering new possibilities for non-destructive biomolecule detection and opening new avenues for identifying TCM extracts. Table 1 summarizes the applications of different spectral analysis techniques in the identification of food-medicine homologous herbal materials.

### 3.3. Methods for DNA Barcoding Identification

**Figure 11:**
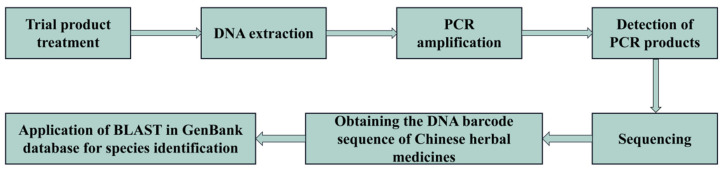
Molecular Identification Process of DNA Barcoding.

Hebert et al. introduced the concept of DNA barcoding in 2003 [135], utilizing short, standardized DNA sequence fragments to rapidly and accurately identify species with high repeatability. These sequences are highly conserved within species but exhibit significant interspecies variation. As the core carrier of genetic information, DNA remains unaffected by external environmental factors, developmental stages, or tissue specificity, effectively addressing misidentifications caused by morphological similarities in traditional taxonomy and errors arising from the instability of chemical characteristics. The molecular identification process for herbal medicine DNA barcoding is illustrated in Figure 11, including DNA extraction, PCR amplification, sequencing, and database comparison to ensure the accuracy of species identification. DNA barcoding for the identification of medicinal plant materials can be categorized into ribosomal DNA barcodes, chloroplast DNA barcodes, mini DNA barcodes, and combinations of these barcodes, based on the selected DNA sequences. Gene fragments, such as matK (megakaryocyte-associated tyrosine kinase), rbcL (ribulose-1,5-bisphosphate carboxylase), ITS (internal transcribed spacer), 5S-rRNA (5S ribosomal RNA), and 18S-rRNA (18S ribosomal RNA), have been successfully utilized as barcodes for this purpose [136]. DNA barcoding is now extensively applied not only in the authentication of medicinal materials [137,138] but also in determining their sources and phylogenetic relationships [139,140].

#### 3.3.1. Ribosomal DNA Barcoding

**Figure 12:**
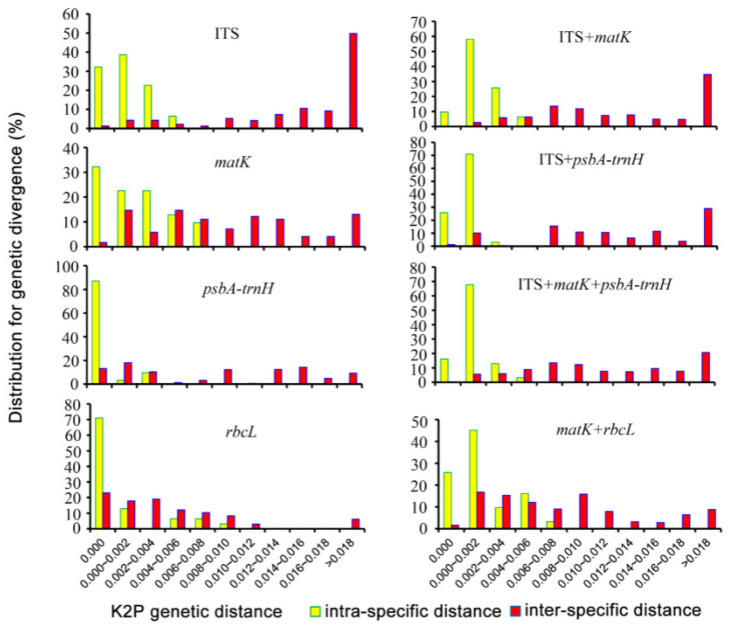
Distribution of the intra- and interspecific variations of the four loci and four regional combinations in Cymbidium species [142].

The Internal Transcribed Spacer (ITS) is one of the most frequently utilized sequencing loci for molecular studies of herbal medicines, especially at lower taxonomic levels, such as genus, species, and subspecies [141]. The ITS region includes the ITS1 intergenic spacer, ITS2 intergenic spacer, and the 5.8S gene (ITS1-5.8S-ITS2), ranging from 400 to 1000 bp. Located within the highly repetitive ribosomal DNA, ITS is characterized by a rapid evolutionary rate, short sequence length, high interspecific variability, and intraspecific consistency. These attributes have made it a widely used genetic marker in angiosperm lineage analysis and fungal metabolite studies. Chen et al. [142] employed DNA barcoding for molecular identification of *Orchidaceae* species in the genus *Cymbidium* and constructed a phylogenetic tree based on the ITS sequence. Figure 12 illustrates the distribution of intra- and interspecific variation for four loci and four region combinations within *Cymbidium* species. Compared to matK, psbA-trnH, and rbcL, the ITS region exhibited only minimal variation in both interspecific and intraspecific comparisons. Among the region combinations, ITS + psbA-trnH showed the most significant variation, indicating its particular effectiveness in distinguishing species. In contrast, the combination of matK + rbcL exhibited the greatest overlap, suggesting that this combination fails to highlight sufficient intra- and interspecific differences, thereby limiting its discriminative capacity as a DNA barcode.

In 2010, ITS2 was introduced as an efficient barcode for medicinal plants, incorporating seven DNA regions from both chloroplast and nuclear genomes to identify medicinal plants and their adulterants [143]. Its ease of amplification and high variability make ITS2 particularly effective for distinguishing closely related species with high accuracy. The ITS2 region is regarded as the optimal and most widely used single-locus barcode for identifying herbaceous plants [144]. Yu et al. [145] applied ITS2 barcoding technology to differentiate *Bupleurum chinense* from *Bupleurum scorzonerifolium*. DNA was extracted using a plant genomic DNA extraction kit, and the ITS2 region was amplified with universal primers. Sequences were assembled using CodonCode Aligner software (https://www.codoncode.com/aligner/, accessed on 9 February 2025), and alignment and haplotype analyses were performed using MEGA 5.0 software. Genetic distances were calculated based on the K2P model. The results indicated that the maximum intraspecific K2P distance for *Bupleurum chinense* was 0.013, while the minimum interspecific K2P distance between *Bupleurum chinense* and *Bupleurum scorzonerifolium* was 0.049, highlighting significant genetic divergence between the two species. However, DNA barcoding based on ITS2 is inadequate for identifying heavily processed herbal materials. To overcome this limitation, Wang et al. [146] developed a short barcode targeting processed *Angelica sinensis* materials. This barcode, designed around a specific single nucleotide polymorphism (SNP) unique to *Angelica sinensis*, was synthesized as a 37-bp nucleotide signature (5′-aatccgcgtc atcttagtga gctcaaggac ccttagg-3′) and effectively identified counterfeit *Angelica sinensis* samples. Furthermore, ITS2 is unsuitable for identifying edible and medicinal ferns [147]. A major issue lies in the presence of multiple copies of ITS2, resulting in substantial intraspecific and even intra-individual sequence variation [148]. Additionally, heterogeneity caused by concerted evolution poses a challenge for ITS2, potentially leading to inaccurate or misleading results.

#### 3.3.2. Chloroplast DNA Barcoding

The psbA-trnH barcode is one of the fastest-evolving regions in the chloroplast genome, situated between the two trnH (H-GUG) sequences and the flanking regions of the psbA gene. Typically, psbA-trnH demonstrates strong primer universality, a high amplification success rate, and an optimal sequence length, making it suitable for low-level taxonomic classification of plants. When compared with nine other genetic markers, including matK, rbcL, and ITS, its discriminatory efficiency was found to be 83%, with a 100% amplification success rate. As one of the fastest-evolving regions in plant chloroplasts, the matK gene is approximately 1500 bp long [149]. Wang et al. [150] employed the matK sequencing method in their analysis of *Desmodium* (*Fabaceae*, *genus Leucaena*), summarizing the advantages and limitations of this technique in DNA barcoding applications for this plant. Total DNA was extracted from nine *Desmodium* samples of different origins using an improved CTAB method, followed by amplification of the matK gene sequence with universal primers specific to *Fabaceae* plants. The results from K2P genetic distance calculations and the construction of the NJ tree indicate that the ITS2 sequence can serve as a DNA barcode for *Fabaceae* plants. The sequence length ranges from 889 to 895 bp, and the genetic distance between different plant species is significantly larger than the largest intraspecific genetic distance. Therefore, the matK sequencing technique can also be used as a DNA barcode for Fabaceae plants, although it exhibits relatively low species resolution. The rbcL barcode is capable of distinguishing plants at the family and genus levels. Its notable advantages include high primer universality, ease of amplification, and robust discriminative ability. However, it is not suitable as a standalone candidate barcode because its slow evolutionary rate limits its capacity to differentiate species. As a result, it is usually combined with other barcodes. In the context of industrial chain applications, Xin et al. [151] used rhubarb as a model and collected 110 samples, including leaves, seeds, roots, decoction pieces, and proprietary Chinese medicines, covering the upstream, midstream, and downstream stages of the rhubarb industrial chain. By selecting the ndhF-rpl32 fragment as a specific DNA barcode, they successfully amplified and sequenced all samples in both directions. The results demonstrated that the ndhF-rpl32 fragment effectively differentiated the seven rhubarb species collected upstream. Among the midstream and downstream samples, 25% of the 36 commercial decoction piece samples were identified as adulterated, whereas all eight proprietary Chinese medicine samples were confirmed to be genuine rhubarb. This suggests that DNA barcoding is a robust and effective technology for traceability across the entire Chinese medicine industrial chain, thereby ensuring the safety of clinical applications.

#### 3.3.3. Micro DNA Barcoding

**Figure 13:**
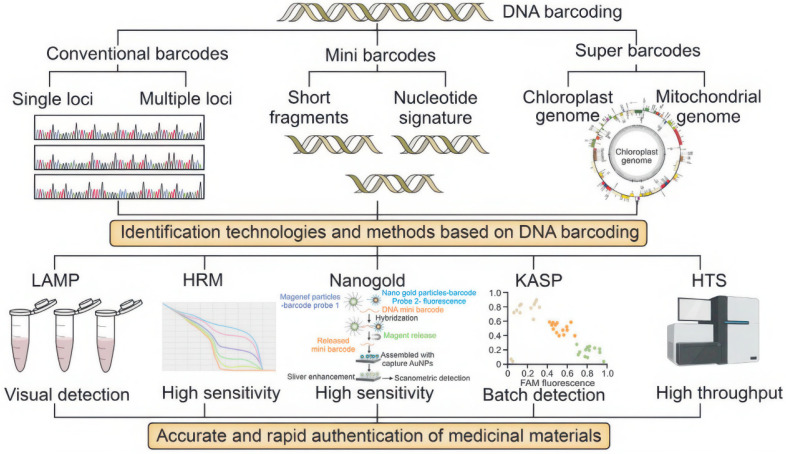
Application of DNA barcoding and its derivative technology in the identification of medicinal resources. Different colored HRM curves are used to distinguish different samples or species. Different colored KASP points are used to differentiate various genotypes or alleles. By analyzing the positions of these curves and points, as well as the shapes of the lines, the specific genotype of a sample can be determined, enabling species identification or variety differentiation [66].

Due to the common phenomenon of DNA degradation in traditional Chinese medicines, obtaining full-length sequence data using conventional standard barcodes is often challenging. Yeo et al. [152] first proposed the concept of a “DNA mini-barcode”, which utilizes molecular markers from 100–300 bp segments to address this issue. Mini-barcodes rely on incomplete, relatively short sequences from standard DNA barcodes to identify different species, making them particularly useful for degraded DNA preservation. One of the most commonly used mini-barcoding regions is the trnL (UAA) intron. The P6 loop of the chloroplast trnL (UAA) intron can be robustly amplified using highly conserved primers, even from degraded DNA samples. Song et al. [153] investigated 45 processed Chinese herbal medicine samples from 15 species and found that only 8.89% and 20% successfully amplified the primary nuclear gene locus ITS2 and four chloroplast gene loci (psbA-trnH, rbcL, matK, and the trnL (UAA) intron), respectively. In contrast, the P6 loop of the trnL (UAA) mini-barcoding region achieved a 75.56% success rate in amplifying processed herbal medicines. This represents a significant improvement over traditional markers and is more cost-effective, as only short DNA fragments are required. In addition to being amplifiable from degraded DNA, mini-barcodes, due to their shorter molecular markers, can be combined with various physicochemical techniques for rapid herbal sample identification, as shown in Figure 13 [66]. The mini-barcode technology, when combined with techniques such as gold nanoparticles, loop-mediated isothermal amplification (LAMP), high-resolution melting (HRM), and high-throughput sequencing (HTS), not only enhances the detection capability of active ingredients in traditional Chinese medicinal materials but also improves the efficiency and accuracy of the identification process. This provides strong technical support for the authentication, quality control, and large-scale identification of traditional Chinese medicinal materials. For example, the combination of mini-barcodes with high-resolution melting (HRM) analysis has been successfully used to accurately identify *Hippophae* (sea buckthorn) in herbal products [154]. Currently, miniature molecular markers are widely applied in quality control for food, supplements, and traditional Chinese medicine products [155]. However, compared to normal barcode regions, designing universal primers for mini-barcodes is relatively challenging, reducing their general applicability. Moreover, the smaller size of mini-barcodes, which contains fewer genetic information and variable sites, inevitably results in lower species resolution. Mini-barcodes often identify samples to the genus level rather than the species level. To maximize the potential of mini-barcodes, it is essential to expand reference databases by adding more sequences.

#### 3.3.4. Super Barcoding and Metabarcoding

Traditional DNA barcoding often faces limitations when distinguishing between closely related species or different populations within the same species. These limitations primarily stem from the slow evolutionary rates of certain genetic markers, such as rbcL and matK, which result in insufficient variation within species. Additionally, the universal primers used in traditional barcoding methods may not effectively amplify DNA across all plant taxa, leading to lower amplification success rates and reduced data reliability. To address these challenges, scholars introduced the concept of the “super barcode” in 2008, which involves sequencing the entire plastid genome as a barcode [156]. The super barcode significantly enhances the accuracy, resolution, and robustness of species identification by integrating multiple genetic markers. It overcomes the limitations of traditional DNA barcoding in distinguishing closely related species with minimal interspecific variation, providing a more powerful tool for resolving complex species groups. For instance, the super barcode has been shown to successfully differentiate closely related species, such as those in the genera *Agathis* (Araucariaceae) [157] and *Echinacea* (Asteraceae) [158], especially in taxonomically challenging groups like the genus Camellia (Theaceae) [159] and the medicinal herb *Epimedium*. The main challenges associated with the super barcode include its high cost, the need for high-quality and abundant DNA, and the large amounts of next-generation sequencing data to process [160]. As the number of plastid genomes in GenBank continues to increase and sequencing technologies advance, the super barcode may become a viable method for species and population-level identification of medicinal plants in the future.

Metabarcoding refers to the simultaneous detection and analysis of DNA barcode sequences from multiple species or individuals within a sample, enabling species population or ecosystem-level identification and analysis. Unlike traditional single DNA barcoding, metabarcoding utilizes high-throughput genomic sequencing technologies to obtain information on multiple species from environmental samples, such as soil, water, or air, in a single analysis [161]. This technique is widely applied in fields such as biodiversity monitoring, species diversity assessment, and ecosystem health evaluation. The core advantage of metabarcoding lies in its ability to overcome the limitations of traditional barcoding methods, particularly in addressing the complexity and diversity of environmental samples, which is crucial for verifying the authenticity of medicinal plant products [162]. However, despite its significant potential, the application of metabarcoding faces some challenges. DNA extraction from complex environmental samples often results in considerable “background noise” from non-target or degraded species, interfering with the detection of target species. Therefore, removing this background noise remains a major challenge for this technology. Additionally, the large volume of data generated by metabarcoding and the inherent errors in high-throughput sequencing present further obstacles. Seethapathy et al. [163] used DNA metabarcoding analysis to evaluate 79 *Ayurvedic* herbal products on the European market, finding that two of the 12 single-ingredient products contained only one species as indicated on the label. Among the 27 multi-ingredient products, only eight species were identifiable, and none were listed on the label. This study underscores the suitability of DNA metabarcoding as an analytical method for identifying complex multi-ingredient herbal products. Urumarudappa et al. [164] identified 39 Thai herbal products listed in Thailand’s National List of Essential Medicines (NLEM), showing that the nuclear region ITS2 enabled the identification of herbal ingredients at the genus and family levels in 55 and 63% of cases, respectively. The chloroplast gene rbcL has been reported to facilitate genus- and family-level identification in 58% and 73% of cases, respectively.

#### 3.3.5. DNA Barcode Combination Method

Studies have shown that relying on a single genetic marker for medicinal plant identification can lead to different results when distinguishing closely related species. A single genetic locus barcode is often insufficient to provide the necessary evolutionary variation to differentiate between these species. Therefore, relying solely on one genetic marker for accurate species identification is not feasible. The advantage of using multiple barcode markers is that the results can mutually validate and complement each other, improving resolution and enabling the differentiation of more species. At an international conference, the CBOL Plant Working Group proposed that the combination of matK and rbcL is suitable for plant barcoding, as matK offers high resolution but low universality, while rbcL provides high universality but lower resolution. Research has shown that the combination of these two barcodes successfully identified 550 species out of 907 samples, with a success rate of approximately 72%. Chen et al. [142] were the first to propose the ITS2 sequence as a universal barcode for identifying medicinal plants, and to combine ITS2 with psbA-trnH as a DNA barcode system for medicinal herb identification. In 2019, five of the top ten medicinal herbs and processed products exported from China were identified using multi-locus barcodes: *Hunan Pinellia* (matK + rbcL), *Ginseng* and *Rhubarb* (psbA-trnH + ITS), *Jujube* (ITS2 + psbA-trnH), and *Angelica* (ITS + rbcL + matK + psbA-trnH) [165]. This further demonstrated the reliability and accuracy of DNA barcode identification methods. Compared to using a single DNA barcode, the combination of multiple DNA barcodes significantly enhances species resolution. However, researchers must determine the optimal combination of DNA barcodes for each application. Using DNA barcode combinations for species identification is more complex and costly than using a single barcode. Therefore, the appropriate identification method should be selected based on the species in question to achieve quick and accurate results.

**Figure 14:**
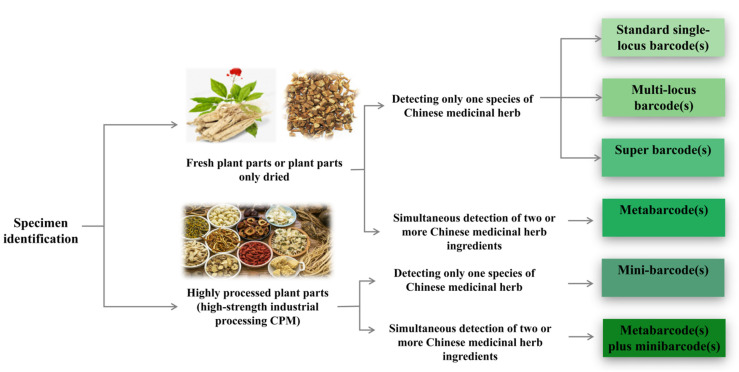
A schematic diagram showing the process involved in choosing the appropriate DNA barcoding technology for the Chinese herbal medicine identification.

The DNA barcode identification methods discussed above exhibit variability in species identification accuracy and applicability for the molecular identification of medicinal plant materials. Selecting an appropriate DNA barcode is crucial for ensuring accurate identification results, as it depends on the specific research requirements and sample type. Figure 14 illustrates the selection of different DNA barcodes for various identification scenarios.

DNA barcoding offers significant advantages in identifying traditional Chinese medicine (TCM) materials. It overcomes the limitations of traditional morphological identification methods, particularly when samples lack distinctive features or are damaged, by providing reliable and reproducible results. Moreover, DNA barcoding is highly effective in distinguishing species with similar morphological traits, offering substantial universality and repeatability. Table 2 presents several additional applications of DNA barcoding. However, challenges persist, especially with samples containing multiple species or those that have undergone extensive processing, which can lead to DNA degradation or contamination. Although current optimization strategies have improved the accuracy of DNA barcoding, further research is needed to address challenges posed by novel processing techniques or specific species identification. Future advancements should focus on enhancing barcode sensitivity, developing efficient DNA extraction and amplification methods tailored to diverse TCM processing conditions, and improving database management and algorithmic optimization to strengthen the stability and applicability of DNA barcoding in TCM.

## 4. Problems and Prospects

### 4.1. Existing Problems

Although significant progress has been made in identifying Food-Medicine Homologous Herbal Materials, with improved accuracy and efficiency achieved through various analytical methods, several challenges and limitations still require further research and resolution. The main challenges include:

(1) Insufficient Datasets and Standardization Affecting Comprehensive Analysis of Medicinal Materials.

Due to the diversity and complexity of medicinal materials, the chemical composition and physical properties of the same material may vary depending on factors such as origin, growing environment, and harvest season. For example, soil rich in calcium and magnesium minerals may promote the synthesis of certain active compounds, while acidic or alkaline soil may affect the absorption of trace elements by plants. A colder environment may enhance the accumulation of certain cold-resistant secondary metabolites, such as polyphenols and flavonoids, whereas high temperatures and drought conditions may lead to changes in essential oils or volatile compounds. Additionally, plants grown at high altitudes typically have higher antioxidant content. posing significant challenges to constructing a comprehensive database. Furthermore, the collection, processing, and storage of medicinal materials lack standardized procedures, hindering unified management and quality control, thereby affecting the comparability and consistency of the data. Although a series of standards have been established at both international and national levels, such as the Chinese Pharmacopoeia, the United States Pharmacopeia, and the World Health Organization (WHO) quality standards for traditional medicinal materials, significant differences remain in policies governing the cultivation standards of Chinese herbal medicines due to cultural and historical variations. These standards include adherence to GACP (Good Agricultural and Collection Practices) for selecting the optimal harvesting time, optimizing processing techniques (such as low-temperature drying, fine grinding, and extraction) to preserve active ingredients, and following GMP (Good Manufacturing Practices) to control storage conditions (such as temperature and humidity management, and light-proof sealing) to extend the stability and shelf life of medicinal materials. However, regional policy differences have posed challenges to the international trade of Chinese herbal medicines. Currently, most of the data are collected by researchers themselves and are often affected by errors and noise, thereby reducing model accuracy and generalizability. Consequently, the true characteristics of medicinal materials may not be reflected accurately, increasing the risk of overfitting.

(2) Multiple Factors Constrain the Improvement of Identification Accuracy.

Chromatography–mass spectrometry (LC–MS/GC–MS) may introduce errors during the preparation of herbal medicine samples, mainly due to sample pretreatment, matrix effects, adsorption and degradation, solvent and mobile phase influences, and systematic errors of the instrument. Hyperspectral imaging can be affected by temperature and humidity changes, compromising its stability and reproducibility. Near-infrared spectroscopy relies on a stable light source, where fluctuations in the light source significantly impact measurement accuracy. Terahertz spectroscopy is susceptible to noise and light scattering, and the system’s sensitivity and resolution limitations may degrade the quality of spectral images. DNA barcoding relies on reference sequence databases, and the lack of barcode sequences for certain species restricts identification capability. These factors may lead to classification errors concerning the variety, authenticity, or origin of medicinal materials of Food-Medicine homology, which could disrupt market order and pose risks to consumer health.

(3) Equipment Cost and Portability Limit the Scope of Application.

Chromatography and mass spectrometry techniques (such as HPLC, GC–MS, and LC–MS) are widely used in medicinal material analysis, but their high costs in equipment acquisition, maintenance, and consumables, especially for mass spectrometry, along with the need for skilled personnel for sample preparation and data analysis, may limit their adoption in small to medium-sized enterprises or resource-limited regions. Near-infrared spectroscopy (NIR) and hyperspectral technology offer advantages such as non-destructive analysis and high efficiency; however, their expensive equipment and the need for specialized calibration and data processing make them more suitable for well-funded research institutions. Terahertz spectroscopy, as an emerging technology, is still in the exploratory stage for medicinal material analysis, and its high equipment and operational costs may hinder broader applications. Although DNA barcoding provides a reliable method for species identification, the high costs associated with high-quality sequencing equipment and reagents, especially when handling large sample volumes, further increase overall expenses. Furthermore, these laboratory-grade analytical instruments are bulky and heavy, with their performance being susceptible to environmental factors like temperature and humidity. These issues constrain their usability in field-based or mobile settings. For situations requiring rapid on-site identification, equipment portability is a critical factor, posing challenges in meeting the efficiency and flexibility of real-world applications.

### 4.2. Outlook

In the future, the methods for identifying food and medicinal materials of the same origin are expected to advance in precision, convenience, and intelligence. The following areas warrant further exploration and development:

(1) Construction of Standardized Datasets for Comprehensive Analysis of Traditional Chinese Medicinal Materials.

To enhance the precision and standardization of identification methods for food and medicinal materials of the same origin, constructing a comprehensive standardized dataset would provide researchers with a reliable and consistent reference framework, thereby promoting systematic studies on the composition, efficacy, quality control, and safety of medicinal materials. By integrating diversified datasets from different varieties, origins, and growth years, including spectral characteristics and DNA barcode feature databases of food and medicinal materials of the same origin, a comprehensive analysis of these materials could be facilitated. As these datasets become more complete and mor standardized, it will enable more accurate pattern recognition, predictive model development, and decision support, further advancing innovation and development in the field of Traditional Chinese Medicine.

(2) Optimizing Multidimensional Factors for Improved Identification Accuracy.

Efforts to develop efficient variable selection and spectral processing methods aim to enhance the performance of quantitative analysis models for medicinal materials by minimizing interference from redundant data and nonlinear factors. Simultaneously, targeted optimization of quantitative analysis models, guided by the spectral characteristics of medicinal materials, could enhance the accuracy and reliability of content detection in mixtures of medicinal materials. Additionally, future research could explore integrating terahertz metamaterials to surpass traditional diffraction limits, potentially enhancing detection speed and accuracy. By leveraging the high sensitivity of terahertz sensors to changes in the dielectric properties of the environment and measuring resonance peak variations (including redshift, blueshift, and offset phenomena) in terahertz spectra based on the unique characteristics of different medicinal materials, this approach could provide distinct spectral signatures for various types of medicinal materials or substances. The observed frequency shift patterns enable precise identification of substances with varying compositions. Combined with accurate quantitative analysis models, this integrated approach holds promise for enabling highly precise and sensitive detection of medicinal materials.

(3) Development of Low-Cost, Portable, and Real-Time Detection Devices.

The development of portable and real-time detection devices in the field of medicinal food homologous material identification is expected to emphasize the integration and innovation of methodologies. These devices should evolve towards multi-functional integration, leveraging advancements in big data, cloud computing, and artificial intelligence to improve the efficiency and accuracy of detection. Future research should prioritize effective preprocessing techniques and streamlined, efficient model architectures to enable real-time detection. Additionally, IoT-based approaches could be integrated to develop intelligent detection systems that collect and transmit data in real time, thereby enhancing both the speed and intelligence of detection processes. The creation of a user-friendly interface combining features such as user and database management into a single platform would facilitate online, real-time, and rapid detection of medicinal food homologous materials, advancing innovative identification methods and fostering comprehensive quality control improvements in medicinal materials.

## 5. Conclusions

Medicinal and edible herbal materials, which offer both medicinal and dietary value, have attracted significant attention in recent years. Accurate identification of the species and multidimensional attributes of these materials is crucial for maintaining market order and safeguarding consumers’ health. Researchers have employed various identification methods, yielding notable results. While traditional methods are simple and convenient, their subjectivity often leads to misjudgments, and they struggle with automation and large-scale identification tasks. With continuous innovation in identification methods, chemical analysis, spectral analysis, and DNA barcoding have provided more scientific approaches for identifying medicinal and edible herbal materials. These methods not only offer higher detection precision and speed but also enable comprehensive external and internal analysis of the materials. However, challenges remain, such as insufficient datasets and lack of standardization, which hinder comprehensive analysis; environmental factors that affect hardware performance, leading to inaccurate identification results; and high equipment costs, which limit widespread application. Furthermore, real-time identification in complex environments remains a significant challenge. This paper provides a comprehensive review of the research progress in modern identification methods, analyzes the issues currently faced by these methods, and offers insights into future development directions, aiming to provide a reference for the innovation and application of identification methods for medicinal and edible herbal materials.

## Figures and Tables

**Table 1:** Applications of Spectral Analysis Methods in the Identification of Medicinal and Edible Herbal Materials.

Image/Spectrum Acquisition Method	Herbal Medicine Name/Category	Method/Model	Result Analysis	Reference
	Fritillaria Zhejiang	CNN, PCA, SVM, PLS-DA	The CNN model achieved accuracy rates of 98.88% on the training set and 88.89% on the test set, indicating the potential advantages of deep learning methods in hyperspectral data processing.	[127]
Hyperspectral Imaging	Goji Berry	PLSR, PCA, IPLS	The PLSR model predicted Vitamin C in the NIR region with an R^2^ value of 0.91. For phenols, SSC, and TA, better predictions were obtained in the VIS–NIR region, with R^2^ values of 0.62, 0.94, and 0.84, respectively.	[128]
	Angelica, Salvia miltiorrhiza	PLS, UPLC	Predicted values for most samples closely matched UPLC-measured values, with trends that were generally consistent, confirming the model’s reliability.	[129]
Near-Infrared Spectroscopy	Cistanche	RF, PLS, PCR, SVM, SG, SNV, MSC	The RF model achieved a calibration set R^2^ of 0.9763 and a RMSE of 0.3527%. For the prediction set, the RF model yielded an R^2^ of 0.9230, RMSEP of 0.5130%, and an RPD of 3.33.	[130]
	Amomum	PCA, PLS-DA, SVM, ResNet	The ResNet model based on synchronized 2D-COS images showed superior generalization and accuracy in identifying Amomum at different drying temperatures.	[131]
	Ginseng	SVM, GA, SPCS, PCA	Through 30 repeated experiments, the SPCS–SVM model maintained high accuracy, while the CS–SVM and GA–SVM models exhibited lower and more fluctuating accuracy.	[132]
Terahertz Time-Domain Spectroscopy	Panax Notoginseng	SVM, LAWOA, 2DCOS, CARS, SVR	The LAWOA–SVM model effectively distinguished Panax Notoginseng from different origins, achieving over 98% accuracy.	[133]
	Flavonoids	KNN, ELM, RF, PLSR, LS-SVM	In qualitative identification, the RF model achieved 100% CCR on the prediction set. In the quantitative model, the LS–SVM model showed better prediction results.	[134]

**Table 2:** Applications of DNA Barcoding Method in the Identification of Medicinal and Edible Herb Materials.

Herbal Medicine Name/Category	Barcode Markers Used	Reference
Ginseng, Vietnamese Ginseng Varieties	ITS2	[166]
Different Species and Sources of Rhubarb	ITS2, psbA-trnH, rbcL	[167]
Fo-ti, Codonopsis	ITS2, psbA-trnH	[168]
13 Varieties of Chinese Sichuan Pepper	ITS2, ETS, trnH-psbA	[169]
Goji Berry	ITS	[170]
Angelica, Chuanxiong, European Angelica	psbA-trnH, ITS	[171]
*Scutellaria baicalensis* and its Adulterants	matK, rpl32-trnL, ndhF-rpl32, trnL-trnF	[172]

## Data Availability

No new data were created or analyzed in this study. Data sharing is not applicable to this article.

## Abbreviations

The following abbreviations are used in this manuscript:

SVM	Support Vector Machine
LDA	Linear Discriminant Analysis
TLC	Thin Layer Chromatography
HPLC	High-Performance Liquid Chromatography
HPLC–ELSD	High Performance Liquid Chromatography–Evaporative Light Scattering Detector
HPTLC	High-Performance Thin-Layer Chromatography
ICP-MS/MS	Inductively Coupled Plasma Tandem Mass Spectrometry
LC–MS	Liquid Chromatography–Mass Spectrometry
HCA	Hierarchical Clustering Analysis
CAMS	Clustering Algorithm based on Multi-Scale
SFA–MS	Sequential Forward Selection–Mass Spectrometry
NIR	Near-Infrared Spectroscopy
FT–NIR	Fourier Transform–Near-Infrared Spectroscopy
GA–PLS	Genetic Algorithm–Partial Least Squares
SNV	Standard Normal Variate
Vis–NIR	Visible–Near-Infrared Spectroscopy
HSI	Hyper spectral imaging
THz	Terahertz
THz–TDS	Terahertz Time–Domain Spectroscopy
OPLS-DA	Orthogonal Partial Least Squares-Discriminant Analysis
CNN	Convolutional Neural Network
PLSR	Partial Least Squares Regression
RF	Random Forest
ResNet	Residual Network
LAWOA	Lévy flight trajectory-based Whale Optimization Algorithm
matK	Megakaryocyte Associated Tyrosine Kinase
rbcL	Ribulose-1,5-bisphosphate carboxylase/oxygenase large subunit
ITS	Internal Transcribed Spacer
PCR	Polymerase Chain Reaction
K2P	Kimura 2-parameter distance
SNP	Single Nucleotide Polymorphism
HRM	High-resolution melting
